# Trends and Public Perception of Artificial Intelligence in Medical Imaging: A Social Media Analysis

**DOI:** 10.7759/cureus.70008

**Published:** 2024-09-23

**Authors:** Mansour Almanaa

**Affiliations:** 1 Radiological Sciences Department, College of Applied Medical Sciences, King Saud University, Riyadh, SAU

**Keywords:** artificial intelligence, medical imaging, public perception, radiology, social media

## Abstract

The rapid advancement of artificial intelligence (AI) in medical imaging has generated significant interest and debate among healthcare professionals, researchers, and the general public. This study aims to explore trends and public perception of AI in medical imaging by analyzing social media discussions. Using a retrospective content analysis approach, social media posts from X (formerly known as Twitter) and Reddit were collected, covering discussions from 2019 to 2024. A total of 1,022 posts were analyzed after data cleaning, employing both qualitative and quantitative methods to examine sentiment, themes, and keyword frequencies. The sentiment analysis revealed that 55% of the comments expressed positive sentiments towards AI in medical imaging, emphasizing its potential to enhance diagnostic accuracy and efficiency. Neutral sentiments accounted for 35% of the posts, while 10% expressed negative sentiments, primarily focusing on concerns related to job displacement, ethical issues, and data privacy. Thematic analysis identified four primary themes: ethical and privacy concerns, job displacement, trust and reliability, and workflow efficiency. Keyword frequency analysis highlighted significant discussions around AI, imaging, and radiology. The results underscore both the optimism and concerns associated with AI in medical imaging, emphasizing the need for ongoing dialogue among technology developers, healthcare providers, and the public. Addressing ethical and privacy concerns, and integrating AI responsibly into clinical workflows, is crucial for maximizing its benefits and minimizing potential risks. These findings provide valuable insights into public perceptions and inform strategies for the effective and ethical implementation of AI technologies in healthcare.

## Introduction

Artificial intelligence (AI) has rapidly transformed various fields, including healthcare, where its application in medical imaging is particularly promising [1-3]. Medical imaging, encompassing modalities such as X-rays, computed tomography (CT), magnetic resonance imaging (MRI), and ultrasound, is a cornerstone of modern diagnostics [4]. AI can assist in various aspects of medical imaging, from image acquisition and enhancement to automated interpretation and diagnosis. By leveraging machine learning algorithms, particularly deep learning, AI systems can analyze vast amounts of imaging data with high precision, potentially identifying abnormalities that might be missed by human observers [1,4,5].

The application of AI in medical imaging has been a focal point of research and development in recent years [6]. Numerous studies have demonstrated the potential of AI to enhance diagnostic accuracy, improve efficiency, and provide new insights into medical conditions through advanced image analysis [1,3-5,7-9]. These capabilities are particularly valuable in fields like radiology, where the volume of imaging studies is continuously increasing, and the demand for timely and accurate diagnoses is critical [10]. One notable application of AI is in the detection of breast cancer through mammography. Research has shown that AI can assist radiologists by identifying suspicious lesions with high sensitivity and specificity, potentially decreasing the rate of false negatives and reducing radiologists' workloads [11-13]. Similarly, AI has been used to analyze MRI images of the brain, aiding in the early detection of neurodegenerative diseases such as Alzheimer's and Parkinson's [8].

Despite the potential benefits, the adoption of AI in medical imaging is not without challenges. Ethical considerations, such as data privacy, algorithmic transparency, and the potential for bias, are significant concerns that must be addressed to gain the trust of both healthcare providers and patients [14]. Additionally, integrating AI into clinical workflows requires substantial changes in practice and training, raising questions about the future roles of radiologists and other medical professionals [14-16].

The advantages and disadvantages of AI in medical imaging have sparked considerable interest and debate among healthcare professionals, researchers, and the general public. Understanding public perception and the specific trends related to AI in medical imaging is crucial for guiding future developments and ensuring the successful integration of these technologies into clinical practice.

Social media comprises various user-driven platforms that facilitate the dissemination of engaging content and discussions with a wider audience, allowing people to express their opinions and ideas [17,18]. Platforms such as X (formerly known as Twitter) and Reddit provide a rich source of data reflecting public and healthcare professionals' perceptions of AI in medical imaging. This study aims to explore the trends and public perception of AI in medical imaging by analyzing social media posts and discussions. The findings provide a comprehensive overview of how AI in medical imaging is perceived by the public and healthcare professionals on social media platforms. By understanding these perceptions, stakeholders can better address the concerns and expectations of different groups, ultimately facilitating the effective and ethical implementation of AI technologies in medical imaging. The results also highlight the importance of transparent communication and the need for ongoing dialogue between technology developers, healthcare providers, and the public to ensure that AI in medical imaging is developed and used in a way that maximizes its benefits while minimizing its risks.

## Materials and methods

Study design

This research utilized a retrospective content analysis design, using both qualitative and quantitative methods to examine social media posts discussing AI in medical imaging. This approach provides a comprehensive understanding of quantitative trends in sentiment and keyword usage, as well as qualitative insights into themes and concerns expressed by social media users. Posts were scraped from two major platforms, X and Reddit, covering discussions from 2019 to 2024. These platforms were selected for their substantial user bases, with X having approximately 550 million monthly active users [19] and Reddit with over 1.2 billion [20]. Additionally, X and Reddit are among the most widely visited websites globally, serving as popular platforms for public discourse and social interaction [21].

Search strategy

Data collection was conducted using a search strategy designed to capture a broad range of discussions related to AI in medical imaging. The keywords used for the search included: "AI," "Artificial Intelligence," "Imaging," "Image," "Medical Imaging," "Computed Tomography," "CT," "Magnetic Resonance Imaging," "MRI," "Ultrasound," "US," "Sonography," "Nuclear Medicine," "Fluoroscopy," "Angiography," "X-ray," "Radiography," "Radiology," and "Radiology Report." Boolean operators "OR" and "AND" were employed to combine these keywords, ensuring comprehensive coverage of relevant topics.

Data cleaning

The data cleaning was performed using Python (version 3.10) [22]. Initially, 1843 posts were collected from X and 2115 from Reddit, resulting in a total of 3958 posts. The data cleaning process involved removing duplicate entries and unrelated posts, such as advertisements. Specifically, 391 duplicate posts and 2545 unrelated posts were excluded, resulting in a final dataset of 1022 posts for analysis.

Sentiment analysis

Sentiment analysis was conducted to categorize the emotions and opinions expressed in the social media posts about AI in medical imaging. This analysis utilized the VADER (Valence Aware Dictionary and sEntiment Reasoner) tool, a sentiment analysis tool implemented in Python that uses a lexicon and rule-based approach, specifically designed to detect sentiments expressed in social media [23]. VADER assigns a compound score to each post, ranging from -1 (most negative) to +1 (most positive). For this study, sentiment scores were categorized into five classes: very positive, positive, neutral, negative, and very negative, by defining specific thresholds for the VADER compound scores.

Thematic analysis

A thematic analysis was conducted manually following Braun and Clarke's (2006) framework [24]. This qualitative approach provided a systematic method for identifying, analyzing, and reporting themes within the data, revealing the main topics and concerns expressed by social media users regarding AI in medical imaging. Four primary themes were identified:

(1) Ethical and privacy concerns, encompassing posts discussing issues related to the ethical use of AI and data privacy; (2) job displacement, involving posts discussing the potential for AI to diminish or replace the roles of radiologists and other medical professionals; (3) trust and reliability, covering posts debating the accuracy and reliability of AI in interpreting medical images and providing diagnoses; (4) workflow efficiency involving discussions on how AI can affect the workload of radiologists and healthcare professionals.

Keyword frequency analysis

Keyword frequency analysis was conducted using Python (version 3.10) [22]. This analysis examined the frequency of specific keywords within the posts to identify the most commonly discussed aspects of AI in medical imaging. By highlighting key trends over time, this analysis provided valuable insights into the focus areas of social media discussions.

## Results

Sentiment analysis

The sentiment analysis of the collected posts revealed a range of emotions and opinions towards AI in medical imaging. The distribution of sentiments over themes is shown in Table 1.

**Table 1:** Distribution of Sentiments Over Themes

Theme	Very Negative	Negative	Neutral	Positive	Very Positive	Total
Ethical and privacy concerns	0	15	60	84	7	166
Job displacement	4	28	126	194	17	369
Trust and reliability	8	21	95	121	11	256
Workflow efficiency	1	21	77	125	7	231
Total	13 (1.3%)	85 (8.3%)	358 (35%)	524 (51.3%)	42 (4.1%)	1022 (100%)

The analysis indicated that the majority of the comments (55%) expressed positive sentiments towards AI in medical imaging, highlighting the benefits of AI in enhancing diagnostic accuracy and efficiency. Examples of positive comments included mentions of AI's ability to assist in the early detection of diseases, aid in interpreting complex images, and reduce the workload of radiologists.

Neutral sentiments made up a significant portion of the comments (35%), providing factual information or posing questions about AI in medical imaging without expressing a clear positive or negative opinion. These comments often discussed technical aspects, requested more information, or shared news about recent advancements in AI technology.

Negative sentiments, including both “negative” and “very negative” categories, comprised a smaller portion of the comments (10%). Concerns raised in these comments included fears about job displacement, ethical issues, data privacy, and the potential for AI to make errors. Users expressed skepticism about the reliability of AI and apprehensions about the loss of the human touch in medical diagnoses.

**Figure 1:**
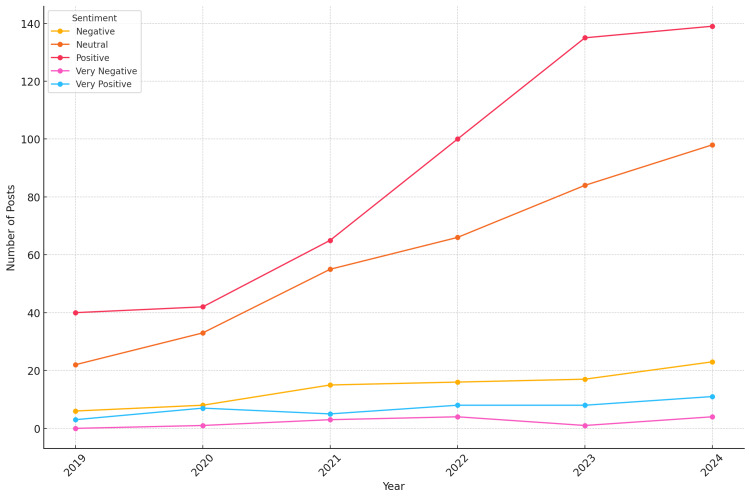
Sentiment Trends Over Time

A line plot depicting sentiment trends over time from 2019 to 2024 showed an increasing trend in positive sentiment, indicating growing acceptance and optimism towards AI in medical imaging (Figure 1).

Thematic analysis

The thematic analysis identified four recurring themes in the discussions about AI in medical imaging. These themes provide deeper insights into the specific areas of interest and concerns of social media users and are discussed in the following sections.

Ethical and Privacy Concerns

**Figure 2:**
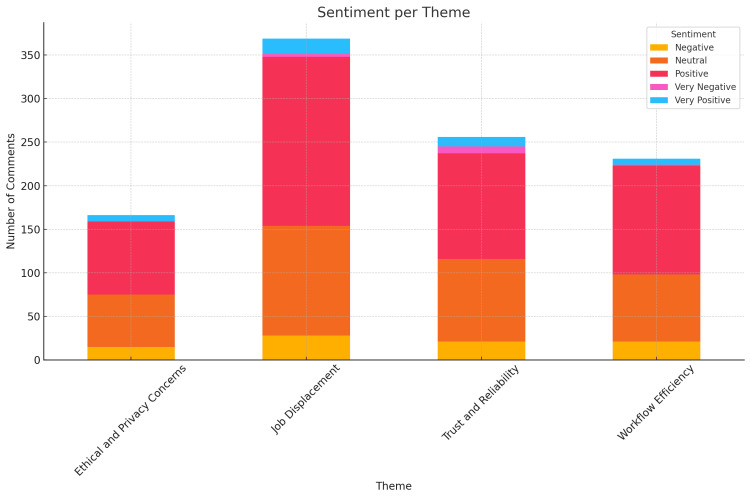
Sentiment per Theme

Ethical and privacy issues were frequently mentioned. Users expressed worries about the security of patient data and the potential misuse of AI technology. Ethical concerns included the transparency of AI decision-making processes and accountability for AI-generated errors. For example, one user commented, “The biggest issue is legal liability. If a human radiologist makes a bad diagnosis, it falls on them and their license. If AI makes a mistake, it falls on the software designer, as well as the hospital which decided to replace human radiology. That can be significantly more expensive as a lawyer could argue negligence on the part of the hospital to rely on such services.” A bar chart showing the average sentiment score by theme highlighted that ethical and privacy concerns yielded a greater number of neutral and negative sentiments compared to other themes (Figure 2).

Job Displacement

The potential for AI to replace human radiologists and other healthcare professionals emerged as a significant concern among social media users. Discussions frequently centered around fears of job loss and the necessity for radiologists to adapt to new technologies. For instance, one user posted, “AI will be able to replace radiologists in a decade.” However, many users expressed the belief that AI would enhance rather than replace radiologists' roles. One user remarked, “AI applications will help radiology job growth and expand radiology applications. It would be a tool to help radiologists, not replace them.” The sentiment analysis, as shown in the stacked bar chart (Figure 2), indicated that the theme of “job displacement” had a substantial number of positive and neutral comments. This reflects a more balanced view of the impact of AI on radiology jobs, with a significant portion of users recognizing the potential for AI to complement and enhance the work of radiologists rather than replace them entirely.

Trust and Reliability

Trust in AI technology emerged as another critical theme. Users frequently questioned the reliability of AI systems and their ability to consistently produce accurate results. Many comments emphasized the necessity for rigorous testing and validation of AI algorithms prior to their widespread adoption. For instance, one user remarked, “The dilemma of AI and data reliability is something that still makes me uneasy about AI. Machine learning is a fantastic technology, and I think we should embrace it, but we have to address the elephant in the room: what if the data we feed it with is incorrect, unreliable, or deliberately wrong?” Despite these concerns, many users expressed optimism that AI will become reliable and trustworthy in radiology in the near future.

Workflow Efficiency

Another prominent theme was the enhancement of workflow efficiency. Users noted that AI could automate routine tasks such as image sorting and preliminary analysis, allowing radiologists to focus on more complex cases and reduce overall workload. An example post stated, “AI has been really helpful, especially during my taster week! Consultants save time and they are able to utilise spare time to teach trainees, perform procedures...” The stacked bar chart (Figure 2) showed predominantly positive sentiments for “Workflow Efficiency.”

Keyword frequency analysis

The keyword frequency analysis provided insights into the most commonly discussed topics related to AI in medical imaging. The top keywords and their frequencies are shown in Table 2.

**Table 2:** Top Keywords and Their Frequencies

Keyword	Frequency
AI	1522
Imaging	884
Radiology	502
Radiologist	304
CT	244

Additional keywords such as "MRI," "diagnosis," "accuracy," "workflow," and "privacy" also appeared frequently, highlighting the diverse range of topics related to AI in medical imaging. These keywords reflect the primary areas of interest and concern among users, aligning with the themes identified in the thematic analysis. For instance, "privacy" was often mentioned in conjunction with ethical concerns, while "workflow" frequently appeared in discussions about efficiency improvements.

**Figure 3:**
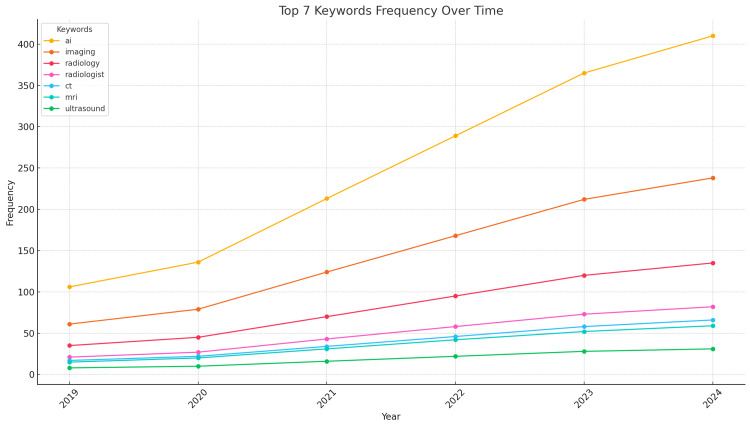
Top Seven Keywords Frequency Over Time

Figure 3 shows the frequency of the top seven keywords over time, illustrating the increasing discussion around "AI" and "imaging" over the years. This trend reflects the growing interest and advancements in these areas.

## Discussion

The analysis of social media posts provides valuable insights into the perceptions and discussions surrounding the use of AI in medical imaging. By examining themes, sentiments, and keyword frequencies, this study reveals both the optimism and concerns expressed by social media users, offering a nuanced understanding of public and professional attitudes toward AI in this critical field.

The sentiment analysis revealed that a majority of the comments expressed positive sentiments towards AI in medical imaging. This optimism is largely driven by AI's potential to enhance diagnostic accuracy, improve efficiency, and reduce the workload of radiologists. Positive sentiments, accounting for 55% of the comments, frequently highlighted the benefits of AI in early disease detection and complex image interpretation. This aligns with the growing body of research demonstrating AI's capabilities in augmenting radiological practices, such as in the early detection of breast cancer through mammography [11-13] and neurodegenerative diseases through MRI [8].

Neutral sentiments comprised 35% of the comments, often discussing technical aspects, seeking information, or sharing recent advancements in AI technology. These comments reflect a cautious yet curious attitude towards AI, indicating that while many users are optimistic, they are also seeking more information to fully understand the implications and applications of AI in medical imaging.

Negative sentiments, although the smallest category at 10%, focused on concerns related to job displacement, ethical issues, data privacy, and the potential for AI errors. These concerns highlight the skepticism and apprehension some users have regarding the integration of AI into clinical workflows. The fear of losing the human touch in medical diagnoses and the potential for AI to make errors that could impact patient outcomes are significant barriers to broader acceptance [14].

The thematic analysis identified four main themes: ethical and privacy concerns, job displacement, trust and reliability, and workflow efficiency. Each theme reflects the primary areas of interest and concern among social media users, providing deeper insights into the specific aspects of AI in medical imaging that are most debated.

Ethical and privacy concerns

The ethical and privacy concerns surrounding AI in healthcare are particularly pressing, especially when patient data is involved. Social media discussions have raised significant issues about how AI systems handle sensitive patient information and the potential risks of data misuse. These concerns are widely recognized in the broader healthcare debate. As AI systems rely on large amounts of data, the need for robust privacy protection and secure data handling becomes crucial. Strong data governance frameworks are essential for maintaining trust in AI applications, particularly in safeguarding patient confidentiality and ensuring compliance with ethical standards [4].

In addition to data privacy, transparency in AI decision-making is a crucial factor for building trust among healthcare providers and patients alike. Lee et al. (2017) emphasize that for AI systems to be fully integrated into clinical practice, medical professionals need to understand how AI reaches its conclusions, ensuring that decisions made by these systems are explainable and reliable [14]. Morley et al. (2021) further underline the importance of interpretability in AI, advocating for models that can be scrutinized and validated by healthcare professionals to prevent errors or biases in decision-making [25]. Without clear ethical guidelines and transparency, AI adoption in healthcare is likely to face significant resistance from both professionals and the public.

Job displacement

Concerns about job displacement due to AI are widespread across various industries, and medical imaging is no exception. Many social media users expressed fears that AI could replace radiologists and other healthcare professionals. While these concerns are understandable, several studies suggest that AI is more likely to complement human expertise rather than replace it. AI has shown the ability to handle routine diagnostic tasks such as image interpretation, but it does not eliminate the need for radiologists. Instead, it enables them to concentrate on more complex and nuanced cases that require critical thinking and human judgment [1]. Similarly, research by Pinto dos Santos et al. (2019) found that medical students are generally optimistic about the integration of AI into radiology, viewing it as a tool to enhance their capabilities and improve diagnostic accuracy, rather than as a replacement for human radiologists [26].

Moreover, other research highlights the transformative potential of AI in radiology, indicating that AI will likely change the role of radiologists rather than render them obsolete. According to Coppola et al. (2021), AI can handle data-heavy processes such as image sorting and preliminary analysis, enabling radiologists to dedicate their expertise to more complex and critical decisions [9]. Likewise, Jha and Topol (2016) suggest that AI will improve radiologists' efficiency by allowing them to focus on tasks that require deep clinical insight, further reinforcing the collaborative role of AI in the healthcare sector [27]. These perspectives, reflected in both the literature and social media discussions, emphasize that continued education and training for healthcare professionals will be critical in adapting to the evolving landscape of AI-driven healthcare.

Trust and reliability

Trust in AI’s reliability remains a significant concern in both public discussions and the academic literature. Many social media users have raised doubts about whether AI systems can consistently deliver accurate and unbiased results, reflecting broader concerns regarding algorithmic bias. This aligns with concerns in the literature regarding algorithmic bias in AI systems. Aggarwal et al. (2021) highlight the risks of biased training data, which could result in AI systems producing inaccurate or harmful outcomes, particularly for underrepresented groups in healthcare [10].

Despite these concerns, the potential for AI to improve its reliability in medical imaging is promising, as advancements in machine learning continue to enhance the accuracy and precision of these systems [7]. With access to more extensive datasets and more sophisticated models, AI can play an increasingly important role in supporting clinical decision-making with high levels of accuracy [28]. As reflected in social media discussions, trust in AI will depend on the continued validation of these systems and the implementation of safeguards to prevent bias and errors.

Workflow efficiency

The discussion around workflow efficiency is a crucial aspect of AI integration in medical imaging, as reflected in both the sentiment analysis and broader academic literature. Many social media users highlighted the potential of AI to streamline routine tasks, such as image sorting, preliminary analysis, and even report generation. The sentiment analysis for this theme showed predominantly positive responses, reflecting a general consensus on the benefits of AI in enhancing productivity. Jin et al. (2021) support these views, noting that AI’s ability to handle repetitive and time-consuming tasks allows radiologists and other healthcare professionals to focus on more complex cases that require human expertise and decision-making [5]. Similarly, Iezzi et al. (2019) argue that integrating AI tools into radiology departments can significantly reduce workloads while improving diagnostic accuracy and reducing errors, leading to better patient outcomes [15].

However, while AI's potential to enhance workflow efficiency is widely acknowledged, it is essential to ensure that these systems are implemented carefully and strategically. Topol (2019) discusses the need for AI to be integrated thoughtfully into healthcare systems, emphasizing that human oversight is crucial, particularly in complex cases. He argues that while AI can significantly enhance efficiency by automating routine tasks, healthcare professionals must remain involved to validate and oversee AI-generated results. Topol stresses that AI should work alongside healthcare workers, enhancing rather than replacing their roles, and ensuring that human judgment is applied in ambiguous or complex medical scenarios [28]. In addition, AI systems should be deployed in a way that aligns with existing clinical workflows, allowing healthcare professionals to leverage these tools without compromising efficiency or accuracy [29]. Furthermore, ongoing training and education for healthcare staff are vital to ensure that AI systems are used optimally. This approach can prevent frustration or inefficiencies that could result from inadequate understanding of AI's capabilities or misalignment with healthcare practices [29]. Overall, the integration of AI can improve workflow efficiency, but its success depends on both the technology itself and the readiness of healthcare teams to adapt to these new tools.

Keyword frequency analysis

The keyword frequency analysis further illuminates how the conversation around AI in medical imaging has evolved. By analyzing frequently used terms, this study revealed the public's major concerns, including issues related to accuracy, workflow, and privacy. Studies in other fields show that keyword analysis can provide real-time insights into public sentiment and highlight emerging trends in technological adoption. Kapoor et al. (2018) argue that analyzing keyword trends in social media discussions helps track the public's understanding of new technologies, as well as identify areas of concern or confusion [17]. In this study, the emphasis on privacy and accuracy reflects the public's ambivalence toward AI, recognizing its potential to enhance efficiency while remaining concerned about its ethical implications.

The increasing frequency of discussions around "AI" and "imaging" over the years indicates a growing interest and recognition of advancements in these areas. This trend reflects the ongoing integration of AI technologies into medical imaging practices and the continuous development of AI capabilities [2,30,31].

Implications for future research and practice

The findings of this study have several implications for future research and practice. The predominantly positive sentiment towards AI in medical imaging suggests a general acceptance of its potential benefits. However, the significant concerns about ethical issues, data privacy, and job displacement need to be addressed to foster broader acceptance and trust.

Future research should focus on developing and implementing robust ethical guidelines and regulatory frameworks to ensure the responsible use of AI in healthcare. Additionally, there is a need for continuous education and training for radiologists and other healthcare professionals to adapt to the evolving landscape of AI technologies.

The balanced view on job displacement indicates that while there is fear of job loss, there is also recognition of AI's potential to enhance professional roles. Therefore, strategies to integrate AI into clinical workflows should emphasize collaboration between AI systems and healthcare professionals, highlighting the complementary nature of AI.

Limitations

This study provides important insights into public perceptions of AI in medical imaging based on social media analysis, but it is important to recognize several limitations. First, the analysis was limited to posts from X and Reddit, which may not fully capture the broader public’s views. The demographic characteristics of users on these platforms, who are often younger and more engaged with technology, could introduce a bias toward more positive or technically informed perceptions of AI. Additionally, the study focused only on English-language posts, potentially overlooking relevant discussions in other languages that might offer different perspectives on AI in medical imaging.

Another limitation relates to the sentiment and thematic analysis methods used. Tools like VADER, while useful for categorizing sentiment, may not accurately detect sarcasm, irony, or more subtle emotional expressions, leading to potential misclassification. Furthermore, thematic analysis, despite being thorough, involves a degree of subjectivity, as the identification and interpretation of themes are inherently influenced by the researcher’s perspective.

Lastly, the study’s timeframe, limited to data from 2019 to 2024, might miss earlier developments or longer-term trends in public perceptions of AI in medical imaging. These limitations suggest that while the findings are valuable, they should be interpreted with caution and in conjunction with further research that includes more diverse data sources and populations.

## Conclusions

The findings of this study provide a comprehensive understanding of public and professional perceptions of AI in medical imaging as reflected through social media discussions. The sentiment analysis highlighted a generally positive outlook, with many recognizing AI's potential to enhance diagnostic accuracy, streamline workflows, and support radiologists in their work. This positive outlook is consistent with the broader narrative within the healthcare community, which increasingly recognizes the transformative potential of AI technologies in radiology and other medical fields. However, the study also identified notable concerns regarding ethical issues, data privacy, and the possible displacement of healthcare professionals, highlighting the complex nature of AI integration in clinical settings.

As AI continues to evolve and its applications in medical imaging expand, addressing these concerns will be crucial for ensuring its responsible and effective use. The identified themes emphasize the importance of developing clear regulatory frameworks and ethical guidelines that ensure patient safety and data security. Equally important is the need for ongoing education and training for healthcare professionals, enabling them to adapt to AI's evolving role in medicine. By fostering a collaborative approach between technology developers, healthcare providers, and policymakers, the medical community can navigate the challenges posed by AI, ultimately leveraging its potential to improve patient care and advance the field of medical imaging.
